# 

**Published:** 1885-04

**Authors:** 


### CONTENTS

ORIGINAL COMMUNICATIONS—

ANNUAL ADDRESS, DELIVERED TO THE GRADUATING CLASS ATLANTA MEDICAL COLLEGE—By Rev. J. B. Hawthorne, D. D, Atlanta, Ga.... 65
ARRESTED AND DEFICIENT LACTATION—By Henry F. Campbell, M. D., Augusta, Ga.... 75
THE TREATMENT OF OVARIAN AND UTERINE TUMORS—By T. J. Word, M. D., Atlanta, Ga.... 81
THE MANAGEMENT AFTER OPERATION, FOR LACERATIONS OF PERINEUM EXTENDING THROUGH THE SPHINCTER ANI MUSCLE—By Geo. H. Noble. M. D., Atlanta, Ga.... 87

SOCIETY REPORTS—

NEW YORK ACADEMY OF MEDICINE.... 89
MACON MEDICAL SOCIETY.... 96

CLINIC REPORTS—

TRACHOMATOUS GROWTHS UPON THE VOCAL CORDS. A CLINICAL LECTURE—By A. W. Calhoun, M. D., Atlanta, Ga.... 98
SYPHILIS AND CHANCROID, A CLINICAL LECTURE—By W. D. Bizzell, M. D., Atlanta, Ga.... 100

EDITORIAL—

THE VALUE PLACED ON EXPERT TESTIMONY by an Atlanta Jury.... 108
OUR PORTRAIT GALLERY.... 110
THE DANGERS OF WATER GAS.... 112
CROWNER'S QUEST LAW.... 115
THE MEDICAL COLLEGES OF THIS CITY, THEIR CLOSING EXERCISES.. 120
MEDICAL ASSOCIATION OF GEORGIA.... 123
THE GEORGIA PRESS ASSOCIATION.... 124
ITEMS.... 128

